# Are school-based behavioural interventions an effective strategy for improving physical activity and sedentary behaviour in children and adolescents? A meta-analysis

**DOI:** 10.3389/fped.2025.1532035

**Published:** 2025-03-11

**Authors:** Liangru Guo, Hengwang He, Chaochao Wang

**Affiliations:** ^1^ School of Sports Science, Hengyang Normal University, Hengyang, Hunan, China; ^2^ School of Physical Education, Shanxi University, Taiyuan, Shanxi, China

**Keywords:** school-based behavioural intervention, children and adolescents, physical activity, sedentary behaviour, 24-h movement behaviour

## Abstract

**Objective:**

This systematic evaluation and meta-analysis [PROSPERO CRD42024598218] was conducted in order to summarise the effectiveness of a body of school-based behavioural interventions on physical activity (PA) and sedentary behaviour in children and adolescents.

**Methods:**

We conducted a systematic search of the literature up to November 2024 using PubMed, Web of Science and SCOPUS. The methodological quality of the included literature was assessed using the Cochrane Risk Assessment Tool.

**Results:**

Of the 6,071 search records initially identified, 26 studies were considered eligible for systematic evaluation and meta-analysis. School-based behavioural interventions were effective in increasing moderate-intensity physical activity [standardised mean difference (SMD), 0.18 (95% CI, 0.04–0.31), *p* = 0.01]. School-based behavioural interventions failed to reduce ST (sedentary time) [−0.04 (95% CI, −0.08 to −0.01), *p* = 0.12] and failed to improve low-intensity PA (LPA) [0.18 (95% CI, −0.07–0.44), *p* = 0.16]. Subgroup analyses showed that school-based behavioural interventions were more effective in improving moderate-intensity PA in children and adolescents who were in school [0.46 (95% CI, 0.20–0.72), *p* = 0.02]. School-based behavioral interventions do not differentiate moderate-intensity physical activity among children and adolescents of different ages, [0.18 (95% CI, 0.05–0.31), *p* = 0.1], nor do they make a difference in moderate-intensity physical activity among children and adolescents in different regions [0.18 (95% CI, 0.04–0.31), *p* = 0.12].

**Conclusion:**

School-based behavioural interventions are effective in increasing moderate-intensity PA among children and adolescents, particularly those who were in school.

**Systematic Review Registration:**

https://www.crd.york.ac.uk/PROSPERO/recorddashboard, PROSPERO [CRD42024598218].

## 1 Introduction

Sedentary behaviours (SB)are typically defined by both low energy expenditure [e.g., resting metabolic rate, typically ≤1.5 metabolic equivalents (METs)] and a sitting or reclining posture (1–3). Sedentary time (ST) is a quantitative indicator of S B and is used to measure the duration of an individual's sitting, reclining, or lying in a low energy expenditure (≤1.5 METs) position (4). Increasing evidence of research demonstrates that both participation in physical activity (PA) and reduction in ST are associated with a number of positive outcomes and benefits to children's health (e.g., self-esteem, well-being, cardiometabolic health, good sleep, etc.) (5). Lack of PA has a significant negative impact on health, contributing to more than 5 million deaths per year globally (6). Excessive ST has been shown to be associated with physical and mental health problems, such as poor body composition, low self-esteem and anti-social behaviours, and reduced academic performance in school-aged children and adolescents (7). Yet globally, more than 85% of children and adolescents do not meet the World Health Organization (WHO) guidelines on PA (8), which suggest that children and adolescents engage in at least 60 min of moderate - to - vigorous - intensity PA (MVPA) daily (8). Additionally, the WHO recommends limiting recreational screen time to no more than 2 h per day to reduce sedentary behaviour and promote healthier lifestyles (9). Therefore, regularly participating in daily MVPA during childhood and adolescence while reducing the chances of ST remains a major challenge in public health (10, 11).

School is an ideal place to promote healthy behaviours in children and adolescents as it is where they spend more than half of their waking hours each day. Children and adolescents have multiple opportunities to be physically active during the last school day, including breaks, sports, physical education classes, and active commuting to and from school. Findings on the impact of school-based interventions on PA levels in children and adolescents have been inconsistent, with school-based PA interventions appearing to have no (12) or only small positive effects (13, 14). Yet other research suggests that interventions targeting these discrete periods of schooling may be effective in increasing children's PA levels (15, 16). Regarding the effectiveness of school-based PA and ST interventions, early studies have mostly relied on self-report measures, which have limited validity and may be differentially biased in subpopulations (17, 18). As research has progressed, more and more studies have begun to use objective measurement tools such as accelerometers to assess PA and ST (1, 19). School-based PA interventions are implemented in a variety of school settings and are often complex, multi-component programmes. It is unclear which school-based strategies are most effective in promoting healthy lifestyles among children and adolescents. Therefore, to fill these gaps in the scientific literature, the present systematic evaluation and meta-analysis aimed to (1) assess the overall effectiveness of school-based interventions (i.e., daily MVPA, ST, and LPA), and (2) investigate the impact of these rates of effectiveness at different ages, in different regions, and during in-school and out-of-school periods.

## 2 Methods

This systematic review is registered with Prospero, the International Prospective Registry for Systematic Reviews (registration number: CRD42024598218). We conducted this systematic review in accordance with the Preferred Reporting Items for Systematic Reviews and Meta-Analyses (PRISMA) statement.

### 2.1 Literature search strategy

The databases PubMed/MEDLINE, EBSCOhost, Cochrane Library and Web of Science (Core Collection) were consulted for literature from their inception to 11 November 2024. In addition, Scopus has been added to ensure a more comprehensive literature search, as Scopus includes a wider range of journals, particularly in the areas of public health and behavioural sciences. A Medical Subject Headings (MeSH) search was performed to establish all relevant literature about this study. In addition, we conducted a reference tracking of published trials and meta-analysis reviews in the field to ensure inclusion of all relevant studies. Specifically, we used the following MeSH terms including “School-Based Services” or “School Health Promotion”. “exercise” or “physical activity” or “training”, “sedentary” or “sedentary time”, and “school-based services” or “school health promotion”, or “sedentary time” or “sedentary lifestyle”, “child” or “child care”, or “children”, “adolescent” or “adolescents”. Detailed search strategies are shown in Supplementary Table S1.

### 2.2 Eligibility criteria

Inclusion criteria were determined according to the PICOS (Population, Intervention, Comparison, Outcome and Study Design) methodology. Studies were included if the following criteria were met: (1) Type of participants: school-aged children and adolescents (5–18 years old). A study was considered eligible if the average age of the participants falls 5–18 years, regardless of the age range of the study samples. (2) Type of intervention: behavioural intervention in a school setting or school-based behavioural interventions included all types of exercise, such as brisk walking, strength training, and yoga. There were no clear requirements for frequency, intensity, or duration of the intervention. Interventions could be categorised as single or multiple group interventions. (3) Type of control group: the control group does not receive any interventions or non-exercise interventions, or receives routine care not involving medical treatment. (4) Type of outcome: mainly including moderate-intensity PA, SB and low-intensity PA. For types of sedentary behaviour, one or more of the following were included: accelerometer-based total sedentary time (assuming ≤100 activity counts as sedentary), self-reported total sedentary time (total sedentary time was used as a proxy measure for total sedentary time in most self-reported methods), screen time (e.g., watching TV, using a computer), occupationally sedentary behaviour (e.g., attending lectures, private study time), or passive transportation) or passive transport. Sedentary behaviours were reported as summary point estimates (e.g., average minutes/hour per day) or as proportions (e.g., percentage of the sample sitting for more than 6 h per day). (5) Type of study design: we included peer-reviewed and English-written randomized controlled trials (RCTs) non-randomized controlled trials, and quasi-experimental designs. Exclusion criteria: (1) Reviews, letters, editorial comments, case reports, conference abstracts, unpublished articles and non-English articles. (2) Studies whose results were not quantified or lacked appropriate outcome indicators. (3) Literature that was not available in full text through all available channels and methods. (4) Articles with poor research quality and no access to quality information. (5) Literature without a control group.

### 2.3 Literature screening and data extraction

All retrieved literature was imported into EndNote software for de-duplication, and then the title, abstract, and full text were read independently by two researchers (LRG and HWH) for literature screening. When disagreements arose, the final results were determined by consensus with the two researchers. Based on the literature screening, the two researchers used a Microsoft Excel spreadsheet to extract and code literature information from the trials. The information for each trial included the first author, country, year of publication, study population, intervention content, intervention protocol (single exercise duration, frequency, and intervention period), measurement tools, and outcome indicators. The methods used to extract the data are described below.

(1) We extracted the mean, standard deviation, and sample size reported for each group before and after the intervention. We combined each outcome indicator using pre- and post-intervention differences (M ± SD). The first step was to calculate the mean difference (the raw mean difference between post-intervention and pre-intervention figures for each intervention group) (20):
  $$MD_{\mathrm{diff}}=M_{\mathrm{post}}-M_{\mathrm{pre}}$$

where, MD_diff_ is the raw mean difference, M_post_ is the reported post-intervention mean, and M_pre_ is the reported pre-intervention mean (20).

$$\mathrm{SD}=\sqrt{N}\frac{CI_{\mathrm{high}}-CI_{\mathrm{low}}}{2t}$$

where, CI_high_ is the upper limit of the confidence interval, CI_low_ is the lower limit of the confidence interval, and t is the t-distribution with N - 1 degrees of freedom in the corresponding confidence interval (20).

(2) The standard deviation of the mean difference (SD_diff_) (20) is calculated as follows:
  $$SD_{\mathrm{diff}}=\sqrt{SD_{\mathrm{pr}e^{2}}+SD_{\mathrm{pos}t^{2}}-2r\times SD_{\mathrm{pre}}\times SD_{\mathrm{post}}}$$

### 2.4 Quality assessment

The Cochrane Risk of Bias Tool is used to assess the quality of eligible trials. The focus was on: (1) whether random sequence was generated; (2) whether the allocation protocol was hidden; (3) whether subjects and staff were blinded; (4) whether the assessment of outcome data was blinded; (5) completeness of outcome data; (6) selective reporting of study results; and (7) other sources of bias. Each item was assessed on a three-tiered scale of bias risk, i.e., low risk of bias, unclear risk of bias, and high risk of bias. Each study was assessed as a whole based on the indicators of the 6 items, which were rated on a three-tiered scale of bias risk, i.e., low risk of bias, moderate risk of bias and high risk of bias, and the risk of bias map was generated by the software Review Manager 5.3. Quality assessment was carried out independently by two researchers, and any disagreements were resolved through discussion with a third person.

### 2.5 Data synthesis and analysis

Evidence synthesis was performed in Review Manager 5.3 (Cochrane Collaboration, Oxford, U.K.). MVPA, LPA, and ST were analysed using continuous variables. All indicators were reported with 95% confidence intervals (CI). Heterogeneity in the study was assessed by the chi-square (χ^2^) test (Cochran's Q) and the index of inconsistency (I^2^) (21, 22). χ^2^ *p* < 0.05 or I^2^ > 50% was considered significant heterogeneity. When significant heterogeneity was detected, a random-effects model was used. Otherwise, a fixed-effects model was applied. Funnel plots were created by Review Manager 5.3 (Cochrane Collaboration, Oxford, UK). Their outcomes were assessed in at least two included RCTs. Sensitivity analyses were applied to the literature of the included studies to test the reliability of the findings. The presence of a significant effect of each article on the combined effect was tested by removing one article at a time.

## 3 Results

### 3.1 Research options

**Figure 1:**
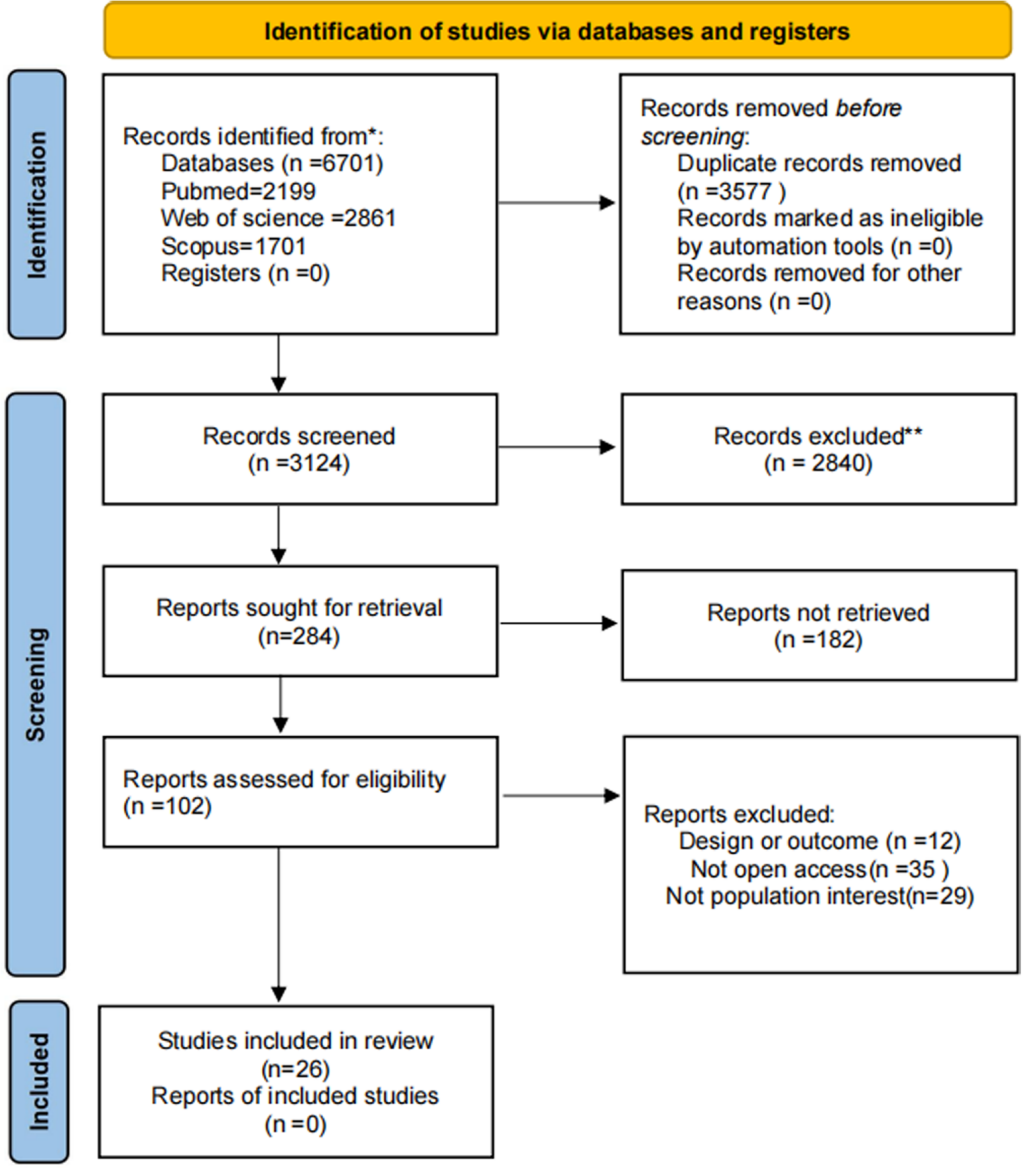
PRISMA 2020 flowchart.

A total of 6,701 studies were identified from the three databases searched. After 3,577 duplicates were removed, 102 full-text manuscripts were identified by screening titles and abstracts. After evaluation of the full text, 76 articles were excluded. Finally 26 articles met the criteria and were included in our systematic review and meta-analysis (Figure 1).

### 3.2 Characteristics of the study

The main characteristics of the participants and interventions are shown in (Table 1). Studies were published between 2005 and 2021, involving 10 RCTs, 4 cluster randomised controlled trials, 2 randomised pilot studies, 2 studies whose types were unclear, 1 pragmatic non-randomized trial, 1 quasi-experimental study, 1 pre-test-post-test design, 1 descriptive study, 1 non-RCT, 1 pilot study, 1 three-arm cluster RCT, and 1 pilot and feasibility study.

**Table 1:** Characteristics of studies included in this meta-analysis.

Study	Country	Sample size	Sex (male, *n*, %)	Age (M ± SD)	Type of intervention	Duration of intervention (weeks)	Outcome	Measuring tools
Christiansen et al. (2021) A pragmatic non-randomized trial	Sweden	IG: 31 CG: 21	IG: 6, 19.4% CG: 11, 42.3%	IG: 16.2 ± 0.5 CG:16.1 ± 0.3	IG: PA-promoting intervention CG: PE	48	MVPA LPA ST	Actigraph GT3X
Kolle et al. (2020) RCT	Norway	IG1: 317 IG2: 288 CG: 229	N/A	IG1: 13.9 ± 0.3 IG2: 14.0 ± 0.3 CG: 14.0 ± 0.3	IG1: PAL IG2: DWBH CG: PE	36	MVPA ST	Actigraph GT3X
Sutherland et al. (2016) RCT	Australia	IG: 645 CG: 505	IG: 312, 48.4% CG: 246,0%	IG: 12 (M) CG: 12 (M)	IG: PA intervention strategies CG: PE	48	MVPA	Actigraph GT3X
Kipping et al. (2014) RCT	UK	IG: 1,024 CG: 1,099	N/A	IG: 9.5 ± 0.3 CG: 9.5 ± 0.3	IG: AFLY5 CG: PE	20	MVPA ST	ActiGraph GT3X+
Ye et al. (2019) A Quasi-experimental study	China	IG: 36 CG: 45	IG: 20, 55.6% CG: 22, 48.9%	IG: 9.42 ± 0.77 CG: 9.09 ± 0.42	IG: exergaming intervention CG: PE	32	MVPA LPA ST	ActiGraph GT3X+
Verstraete et al. (2007) A pre-test–post-test design	Belgium	IG: 243 CG: 243	N/A	11.2 ± 0.7	IG: SPARK programme CG: PE	68	MVPA LPA	ActiGraph GT3X+
Lau (2016) RCT	China	IG: 40 CG: 40	IG: 29, 72.5% CG: 26, 65.0%	N/A	IG: Xbox 360 CG: PE	12	MVPA	ActiGraph GT3X+
Nathan et al. (2020) RCT	Australia	IG: 1,169 CON: 693	IG: 598, 52.1% CG: 331, 47.9%	IG: 7.96 ± 2.03 CG: 8.05 ± 2.05	IG: Physical Active Children in Education CG: PE	36	MVPA	ActiGraph GT3X+
Carlin et al. (2018) A randomised pilot study	UK	IG: 129 CG: 135	N/A	IG: 12.54 ± 0.57 CG: 12.16 ± 0.51	IG: walking sessions CG: PE	12	MVPA LPA ST	ActiGraph GT3X+
Cui et al. (2012) A pilot study	Australia	IG: 346 CG: 336	IG: 176, 50.9% CG: 177, 52.7%	N/A	IG: four-component intervention CG: PE	4	ST	PA questionnaire
Gammon et al. (2019) RCT	UK	IG: 96 CG: 74	N/A	11–14 (range)	IG: PAL training CG: PE	8	MVPA LPA ST	Axivity AX3
Peralta et al. (2005) RCT	Australia	IG: 12 CG: 11	IG: 12, 100% CG: 11, 100%	12.5 ± 0.4	IG: FILA CG: general fitness program + PE	24	MVPA	MTI 7164 Actigraph accelerometers
Schofield et al. (2005) A descriptive study	Australia	IG1: 23 IG2: 24 CG: 21	N/A	IG1: 15.9 ± 0.8 IG2: 15.7 ± 0.8 CG: 15.9 ± 0.8	IG1: GSOP (PED) IG2: GSOP (MIN) CG: PE	16	MVPA	3DPAR
Haerens et al. (2006) RCT	Belgium	IG: 63 CG: 77	N/A	13.1 ± 0.8	IG: school-based intervention programme CG: PE	84	MVPA	Physical Activity Questionnaire (FPAQ)
Seljebotn et al. (2019) A cluster randomized controlled trial	Norway	IG: 228 CG: 219	IG: 118, 52% CG: 108, 49%	9 ± 10 years (range)	IG: outdoor physically active academic lessons CG: PE	40	MVPA LPA ST	ActiGraph GT1M/GT3X/GT3X+
Okely et al. (2017) RCT	Australia	IG: 306 CG: 298	IG: 0, 0% CG: 0, 0%	13.6 ± 0.02	IG: unique 18-month action plans CG: PE	72	MVPA LPA ST	Actigraph accelerometer
Angelopoulos et al. (2009) RCT	Greece	IG: 321 CG: 325	IG: 137, 42.7% CG: 149, 45.8%	IG: 10.25 ± 0.44 CG: 10.29 ± 0.44	IG: school-based nutrition and PA intervention programme CG: PE	48	MVPA	Standardized questionnaire
Azevedo et al. (2014) Non-RCT	UK	IG: 280 CG: 217	IG: 36.1% CG: 35.5%	IG: 11.2 ± 0.4 CG:11.3 ± 0.4	IG: PE + dance mats CG: PE	48	MVPA ST LPA	Actigraph GT3X
Bell et al. (2017) A pilot study	UK	IG: 233 CG: 211	N/A	12–13 years	IG: AHEAD CG: PE	28	MVPA ST	Accelerometer processing decision
Cohen et al. (2015) A cluster randomized controlled trial	Australia	IG: 62 CG: 72	N/A	IG: 8.5 ± 0.7 CG: 8.5 ± 0.6	IG: SCORES CG: PE	48	MVPA	ActiGraph GT3X
Gorely et al. (2011)	UK	IG: 310 CG: 279	N/A	IG: 8.8 CG: 8.9	IG: GreatFun2Run CG: PE	40	MVPA	ActiGraph GT1M
Howe et al. (2014)	USA	IG: 11 CG: 16	N/A	N/A	IG: 30 min of free play daily to all third graders CG: PE	36	MVPA	Actigraph GT1M
Jago et al. (2012) Three-arm, cluster RCT	UK	IG: 100 CG: 130	IG: 0, 0% CG: 0, 0%	11–12 years	IG: after-school dance classes CG: PE	36	MVPA	Actigraph accelerometer
Jago et al. (2) (2012) A cluster randomised feasibility trial	UK	IG: 153 CG: 157	N/A	9–11 years	IG: Action 3:30 CG: PE	20	MVPA	ActiGraph accelerometer
Kriemler et al. (2010) A cluster randomised controlled trial	Switzerland	IG: 297 CG: 205	N/A	6.9 ± 0.3 11.1 ± 0.5	IG: multi-component PA programme CG: PE	44	MVPA	Accelerometer
Masini et al. (2020) A pilot and feasibility study	Italy	IG: 16 CG: 12	N/A	9.02 ± 0.11	IG: active breaks CG: PE	16	MVPA	Actigraph accelerometers

IG, intervention group; CG, control group; MIG, moderate-intensity group; LIG, low-intensity group. The physically active learning (PAL) intervention included 30 min physically active learning, 30 min PA and a 60 min physical education (PE) lesson per week. The Don't worry-Be happy (DWBH) intervention included a 60 min PA lesson and a 60 min PE lesson per week, both tailored to promote friendships and well-being.

The 26 studies were conducted in 9 countries, including 7 (26.9%) in Australia (23–29), 8 (30.8%) in UK (30–37), 2 (7.7%) in China (38, 39), 2 (7.7%) in Belgium (40, 41), 2 (7.7%) in Norway (22, 42), 1 (3.8%) in Sweden (43), 1 (3.8%) in Greece (44), 1 (3.8%) in Switzerland (45), 1 (3.8%) in USA (46) and 1 (3.8%) in Italy (47). A total of 12,464 individuals were included in the studies. A detailed description of the study participants is given in Table 1. The interventions were all school-based behavioural interventions whose durations ranged from 4 weeks (25) to 84 weeks (25). Regarding the types of intervention outcomes, 25 (96.2%) studies reported MVPA (22–24, 26–47), 13 (50%) studies reported ST (22, 24, 25, 28, 30–34, 39, 41–43), and 9 (56.3%) studies reported LPA (28, 31–33, 39–43).

### 3.3 Risk of bias assessment

**Figure 2:**
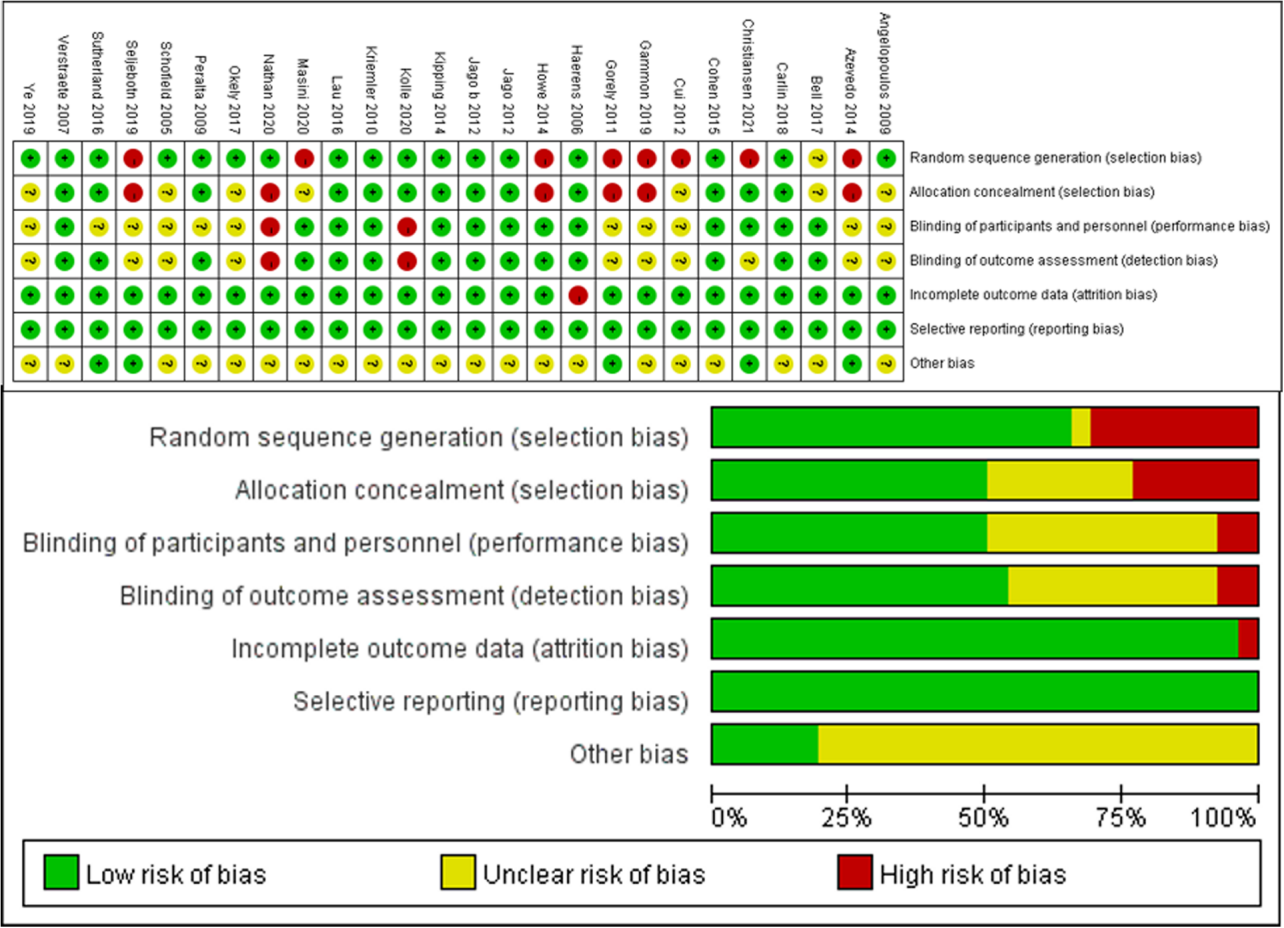
Summary of risk of bias. Risk of bias summary: Review authors’ judgement of risk of bias items for each included study. Below: Risk of bias graph: Review authors’ judgement of each risk of bias item, expressed as a percentage of all included studies.

Figure 2 summarises the risk of bias. Overall, the risk of bias for the 26 trials included in the review was within acceptable limits. Seventeen (65.4%) trials had adequately determined random sequences, and thirteen (50%) adequately implemented allocation concealment. Thirteen (50%) trials blinded participants and staff while 14 (83.8%) were blinded to outcome assessors, and the risk of bias for these trials was judged to be low. In 26 (100%) trials, there were no drop-outs or selectivity reported. Therefore, the risk of bias for these trials was judged to be low.

### 3.4 Results of the meta-analysis

In the included trials, school-based behavioural interventions for PA levels and sedentary behaviour outcomes of children and adolescents were assessed using a variety of tools. In our review, a meta-analysis was conducted focusing on ST, moderate-intensity PA, and low-intensity PA. Change scores from baseline to final values were used in our final efficacy analyses. The results of our analyses for each outcome are presented below.

#### 3.4.1 Sedentary time

**Figure 3:**
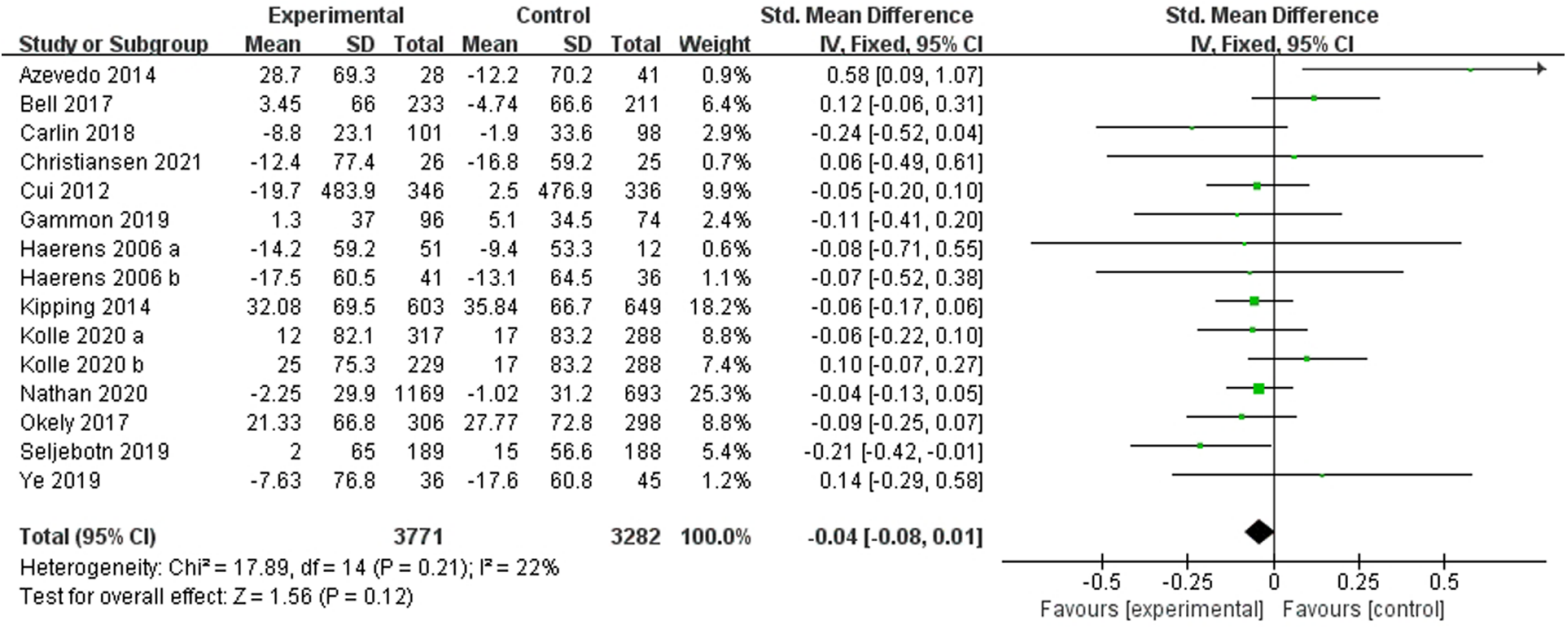
Forest plot of the effect of school-based exercise on ST. CI, confidence interval.

Thirteen (22, 24, 25, 28, 30–34, 39, 41–43) studies reported ST and included 6,765 subjects. One study (22) divided the intervention group into two subgroups with different interventions, and one study (41) divided the subjects into two different groups by gender. Fifteen were therefore included in the meta-analysis, and a fixed-effects model was used due to the small heterogeneity present in this review (I^2^ = 22%). The results showed a combined sample size of 7,053 and a non-significant level of ST for the school-based exercise intervention compared to the control group [SMD = −0.04, 95% CI = (−0.08, 0.01), *p* = 0.12] (Figure 3).

#### 3.4.2 Moderate-intensity PA

**Figure 4:**
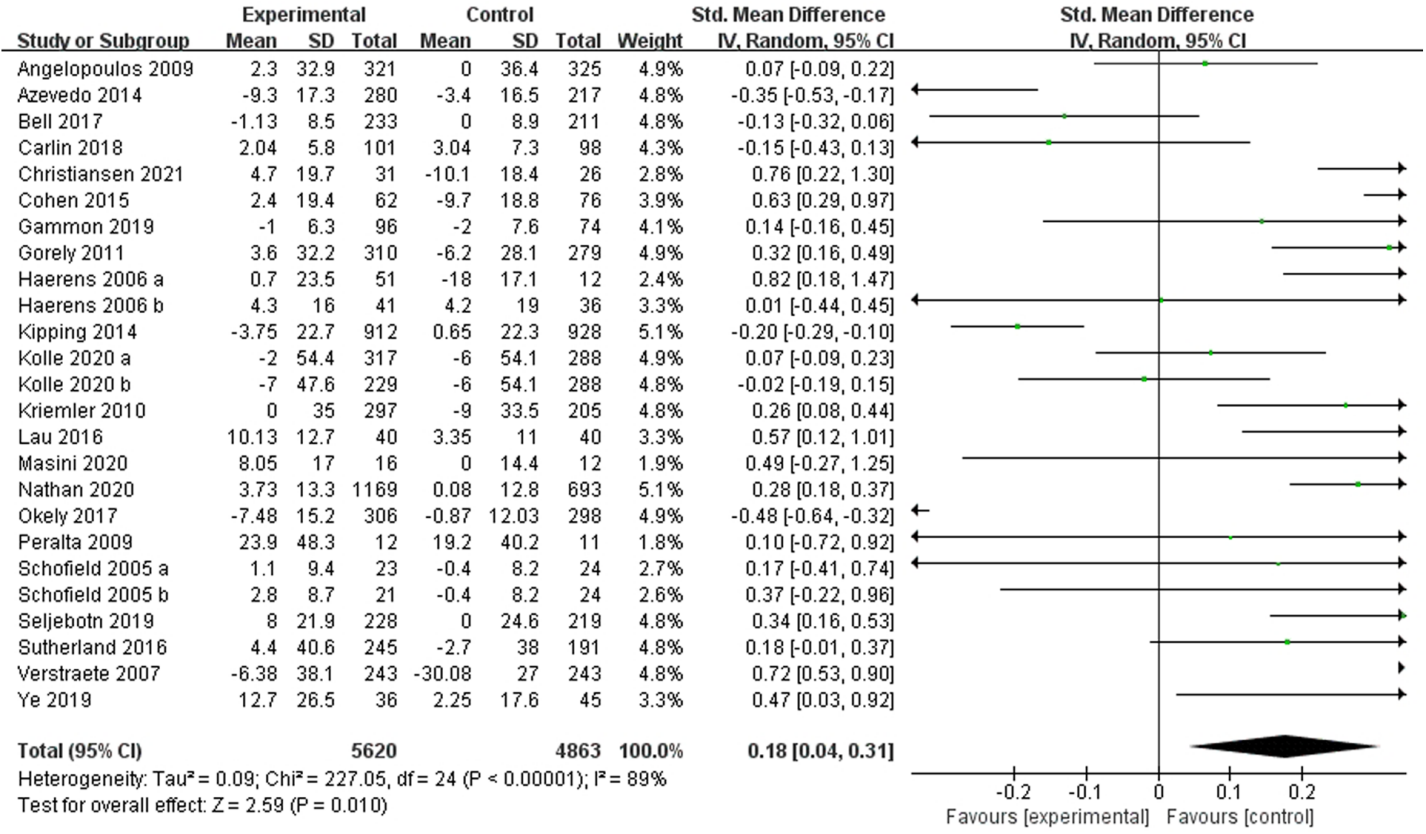
Forest plot of the effect of school-based exercise on moderate-intensity PA. CI, confidence interval.

Twenty-two (22–24, 26–35, 38–45, 47) studies reported moderate-intensity PA and included 10,171 subjects. Two studies (22, 27) divided the intervention group into two subgroups with different interventions, and one study (41) divided the subjects into two different groups by gender. Therefore 25 studies were included in the meta-analysis and a random-effects model was used due to the high heterogeneity present in this review (I^2^ = 89%, *p* < 0.00001). The results showed a combined sample size of 10,483 and evidence of a significant level of exercise intervention (moderate-intensity PA) compared to the control group [SMD = 0.18, 95% CI = (0.04, 0.31), *p* = 0.01] (Figure 4). We further conducted subgroup analyses (Table 2). School-based behavioural interventions were found to be more effective in improving moderate-intensity PA in children and adolescents who were in school compared with those who were not in school. No significant differences were found between age subgroups and between study locations.

**Table 2:** Results of subgroup analysis.

Outcome	Subgroup		*N*	SMD [95% CI]	I^2^	*p* (subgroup)
MVPA	Age (years)	6–12	13	0.26 [0.10, 0.43]	92%	0.1
		12–18	9	0.06 [−0.13, 0.24]	78%	
	Region	Europe	14	0.15 [−0.01, 0.31]	90%	0.12
		Australia	6	0.16 [−0.16, 0.49]	92%	
		Asia	2	0.52 [0.20, 0.83]	0%	
	Whether or not they are in school	In school	10	0.46 [0.20, 0.72]	93%	0.02
		Out of school	2	0.13 [0.02, 0.24]	3%	

#### 3.4.3 Low-intensity PA

**Figure 5:**
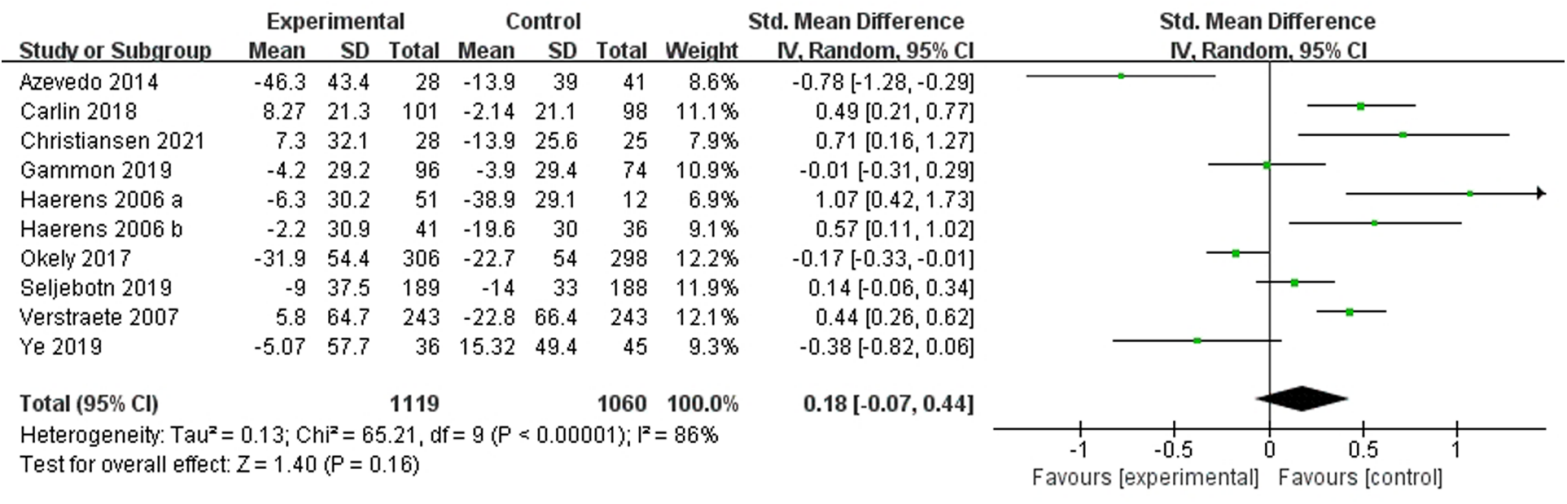
Forest plot of the effect of school-based exercise on low-intensity PA. CI, confidence interval.

Nine studies (28, 31–33, 39–43) reported low-intensity PA and included 2,179 subjects. One study (41) divided subjects into two different groups by gender. Therefore 10 were included in the meta-analysis and a random-effects model was used due to the high heterogeneity present in this review (I^2^ = 86%, *p* < 0.00001). The results showed no evidence of a significant exercise intervention (low-intensity PA) compared to the control group [SMD = 0.18, 95% CI = (−0.07, 0.44), *p* = 0.16] (Figure 5).

### 3.5 Sensitivity analysis

**Figure 6:**
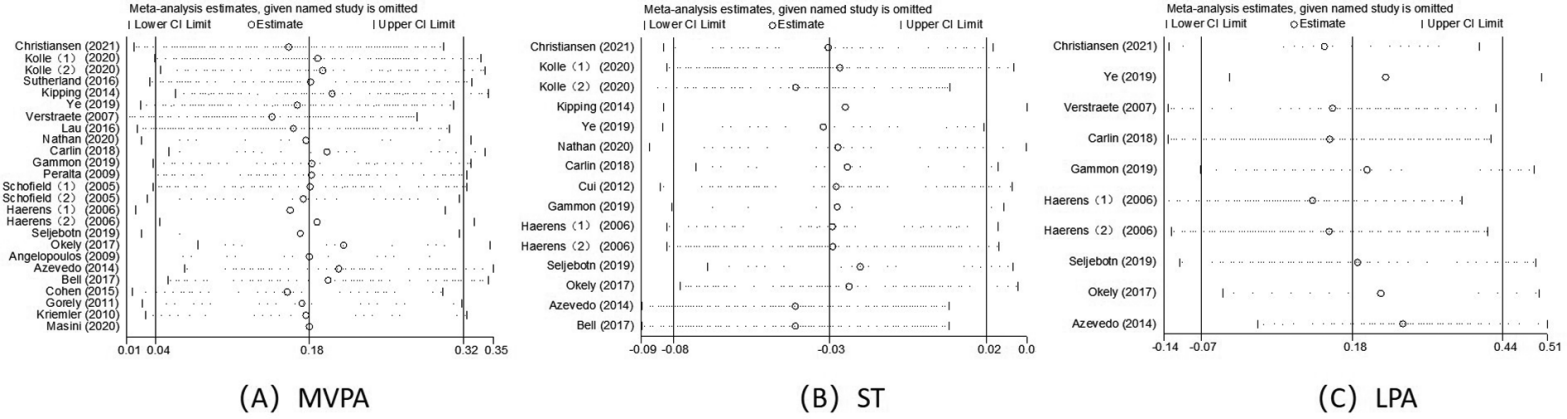
Sensitivity analysis of **(A)** MVPA, **(B)** ST, **(C)** LPA.

Sensitivity analyses were conducted to assess the effect of school-based behavioural interventions on MVPA, ST and LPA in children and adolescents in each study. Figure 6A: The results of the meta-analysis of school-based behavioural interventions on MVPA in children and adolescents were statistically significant, OR (95% CI) = 0.18 (0.04, 0.32). Figure 6B: The results of the meta-analysis of school-based behavioural interventions on ST in children and adolescents were statistically significant, OR (95% CI) = −0.03 (−0.08, 0.02). Figure 6C: The results of the meta-analysis of school-based behavioural interventions on LPA in children and adolescents were statistically significant, OR (95% CI) = 0.18 (−0.07, 0.44). Sensitivity analyses showed good robustness of the results of school-based behavioural interventions on MVPA, ST and LPA in children and adolescents after any separate studies are excluded.

### 3.6 Detection of publication risk of bias

**Figure 7:**
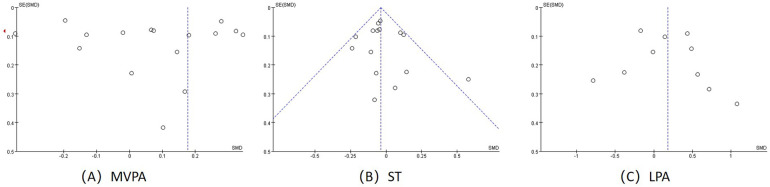
Funnel plots; **(A)** funnel plot for MVPA **(B)** funnel plot for ST **(C)** funnel plot for LPA.

According to the funnel plots of school-based behavioural interventions on MVPA, ST and LPA for children and adolescents (Figure 7), the left and right sides of the funnel plots for ST and LPA are largely symmetrical, with less publication bias, the funnel plots for MVPA are less symmetrical, and therefore Egger's and Begg's tests were performed to further test for publication bias.

We used Egger's and Begg's linear regression tests to examine publication bias (Table 3) (Supplementary Table S1). MVPA (*t* = 1.34, *P* = 0.194), ST (*t* = 0.11, *P* = 0.914) and LPA (*t* = 0.48, *P* = 0.645) were obtained by Egger's linear regression test. The data obtained by Egger's linear regression test met the criterion when there was no publication bias. By Begg's linear regression tset, MVPA (*z* = 0.7, Pr = 0.46), ST (*z* = 0.45, Pr = 0.65) and LPA (*z* = 0.36, Pr = 0.72) were significantly different. The data obtained by Begg's linear regression test all met the criteria when there was no publication bias. Therefore, there was no publication bias in MVPA, ST, and LPA.

**Table 3:** Analysis of bias.

Outcome indicators	Egger		Begg	
	*t*	*P*	*z*	Pr
MVPA	1.34	0.194	0.74	0.46
ST	0.11	0.914	0.45	0.65
LPA	0.48	0.645	0.36	0.72

## 4 Discussion

### 4.1 Main results of the article

Twenty-six studies assessing the impact of school-based behavioural interventions to improve PA and sedentary behaviour in children and adolescents were considered eligible for our systematic evaluation and meta-analysis. We systematically evaluated the available studies and extracted information on sample characteristics, study design, key methodological features, PA and sedentariness. However, most of the included studies were RCTs on exercise interventions and could not be fully blinded. Previous studies have shown that the results of trials using optimal methods may still be at risk of bias. However, it is not reasonable to regard trials as low quality because they were not blinded. During the quality assessment process, we employed the Cochrane Risk of Bias tool (version 1.0) to evaluate the methodological quality of the included studies. This tool was chosen due to its suitability for behavioural intervention research. We did not utilise the GRADE system as our focus was on synthesising effect sizes rather than grading the quality of evidence. The assessment results indicated that 26 studies were deemed to be of high quality, which significantly enhances the credibility and validity of our research, thereby rendering the obtained results and conclusions more accurate and reliable. In order to synthesise the findings on whether school-based behavioural interventions were effective in improving PA and sedentary behaviours in children and adolescents, a meta-analysis of MVPA, ST and LPA was conducted in this study. This meta-analysis provided evidence that school-based behavioural interventions were able to have a smaller but statistically significant impact on moderate-intensity PA in children and adolescents compared to the control group. Subgroup analyses of MVPA, an outcome indicator, by age of the participants, region of study, and time (in or out of school) showed that interventions were more effective in increasing MVPA in children and adolescents during their time in school, with no differences by age or region. School-based behavioural interventions did not improve ST and LPA in children and adolescents.

### 4.2 Overall effect

The school setting has long been defined as the ideal environment for PA promotion interventions. Because children and adolescents spend the majority of their waking hours in the school setting, school-based interventions may be necessary to promote PA in children and adolescents. Our findings update the systematic evaluation of school-based behavioural interventions on PA and sedentary behaviours in children and adolescents. The results of this study suggest that school-based behavioural interventions increased MVPA but had no significant effect on ST and LPA in children and adolescents. This is consistent with the results obtained from a number of previous systematic evaluations, where school-based behavioural interventions have been able to improve PA (48), particularly in urban/suburban schools (49), as well as increase LPA levels and decrease ST in children and adolescents (48, 50). In contrast, some studies have produced opposite findings that school-based behavioral interventions did not improve MVPA (12, 50, 51) and did not improve LPA and ST (51). The inconsistency of the findings is on the one hand attributable to the different results from the included studies. On the other hand, it may be attributed to the different ways (objective accelerometer measurements vs. subjective questionnaires) of assessing PA and ST. Although they have been shown to be valid and reliable in estimating children's PA, accelerometers have the inherent limitation of not being able to take measurements in large numbers, limiting the coverage of whole cohort surveys (52). Moreover, the ability to detect only certain activities and upper body movements, cycling or resistance training may be underestimated (53). In turn, the validity and reliability of questionnaires are relatively weak (54). In addition, these are also sources of potential heterogeneity in the articles and also include differences in intervention design (e.g., duration, intensity, and method of delivery), physical activity, and different characteristics of the study population (e.g., age, gender, and cultural background).

### 4.3 Effects of moderating variables on school-based behavioural interventions on PA

First, the effect of participants' age differences on PA in children and adolescents was assessed. This study involved two subgroups of children (6–12 years old) and adolescents (12–18 years old). The results showed that there were no significant differences between the two subgroups, thus demonstrating that the age of the participants was not a significant factor influencing the effectiveness of school-based behavioural interventions. A meta-analysis concluded that the effects of school-based interventions on PA in older adolescents were usually small and short-term (55). In a specific study that yielded results inconsistent with the present study, the effect size of school-based behavioural interventions increased significantly with the age of the students, with the greatest improvement in the oldest group (Grade 6 students) (24). And a meta-analysis also noted that multiple school-based behavioural interventions were effective in increasing self-reported PA in trials of students aged 13 years or older (48). Given the differences between primary and secondary education, for example, in terms of teaching provision and flexibility of the school day, the resulting studies differed, but the impact of school-based behavioural interventions on PA of children and adolescents was undisputed.

Second, the effect of the region of study on PA of children and adolescents was examined. This study involved three subgroups by the region of study: Europe, Asia and Australia. The results showed no significant differences between the three subgroups, thus demonstrating that the region of study was not an important factor influencing the effectiveness of school-based behavioural interventions. In one study it was noted that a much higher proportion of studies on this topic were conducted in Europe (56). The results of one of these studies suggest that the implementation of interventions in schools in deprived areas has the potential to reduce the decline in PA among adolescents (23). In a study conducted in China, it was noted that Chinese high school students have long school hours (5.4 days of school per week, 7.6 lessons per day 28) and high academic stress (2 h per day for homework) (57). The situation of children and adolescents varies from region to region, but the results of the subgroup analyses show that region does not influence the effectiveness of school-based behavioural interventions.

Thirdly, the impact on PA of children and adolescents in and out of school was examined. The results indicate that the impact on PA was much greater when the participants were in school than that when they were out of school, thus demonstrating that school-based behavioural interventions were an important factor in influencing the PA of children and adolescents in school. No meta-analysis has been conducted to identify the effects of participants on PA when they were in and out of school. The results from one study demonstrated a positive and stronger effect of interventions on PA levels observed during school hours (22). A 10-min increase in MVPA per weekday was equivalent to a 50-min increase in MVPA per week, representing an increase of approximately 30% in PA at baseline (36). School-based interventions were effective in increasing PA levels in children and adolescents.

### 4.4 Limitations and advantages

This systematic evaluation and meta-analysis also had several limitations. First, most of the included studies were randomised controlled trials on exercise interventions and could not be fully blinded. Therefore, subjective factors could cause some degree of bias in the quality assessment process. Second, most studies used accelerometers to assess PA and sedentary behaviour, but a small number of studies used subjective questionnaires for assessment, resulting in high heterogeneity. Third, we combined pre- and post-intervention differential effect sizes and did not consider the long-term impact of the intervention, as only a few of the included studies reported follow-up data in the meta-analysis. This study also has several strengths. First, it is an innovative study since there has not been a systematic evaluation and meta-analysis on the impact of school-based behavioural interventions on PA and sedentary behaviour in children and adolescents, particularly sedentary behaviour. Second, this review employed a rigorous systematic review methodology in accordance with PRISMA guidelines to ensure that relevant literature was identified and assessed with the highest possible scientific rigour. Third, this review provides an *a priori* design for registration in the Prospero database. Therefore, research questions and inclusion criteria were established prior to conducting this review. Fourth, three electronic sources were searched, as reported above. And, we detailed the search strategy in the electronic Supplementary Table S1. In addition, the quality of the included studies was examined, and the conclusions drawn from this review were strengthened through the use of a quality assessment tool. Fifth, the subgroup analyses conducted in the study are representative, and a distinctive viewpoint can be seen from the study. Our data are able to provide support for policy makers and, in real time, initiatives to improve PA and increase positive health outcomes in children and adolescents.

## 5 Conclusions

The present systematic evaluation and meta-analysis suggest that school-based interventions are effective in increasing moderate-intensity PA among children and adolescents, especially during the school days. In future studies, firstly, it is necessary to expand the sample size, standardise the tools for evaluating PA and ST and extend the exercise cycle so that the results of the meta-analysis can be as comprehensive as possible. Secondly, more rigorous and more scientific methods are expected so as to improve the quality of RCTs and draw more rigorous conclusions as much as possible, thus providing better references for relevant medical practitioners as well as physical education teachers.

## Data Availability

The datasets presented in this study can be found in online repositories. The names of the repository/repositories and accession number(s) can be found in the article/Supplementary Material.
