# Supplementary material for: Are school-based behavioural interventions an effective strategy for improving physical activity and sedentary behaviour in children and adolescents? A meta-analysis
Source: Front Pediatr. 2025 Mar 11;13:1532035. doi: 10.3389/fped.2025.1532035 (PMC11933046; doi:10.3389/fped.2025.1532035)
Supplement: Supplementary file 1 [file Datasheet1.docx]

Supplementary Material

# Search string

## **Search string for Pubmed**

("school health services"[MeSH Terms] OR ("school"[All Fields] AND "health"[All Fields] AND "services"[All Fields]) OR "school health services"[All Fields] OR ("health"[All Fields] AND "service"[All Fields] AND "school"[All Fields]) OR "health service school"[All Fields] OR ("school health services"[MeSH Terms] OR ("school"[All Fields] AND "health"[All Fields] AND "services"[All Fields]) OR "school health services"[All Fields] OR ("school"[All Fields] AND "health"[All Fields] AND "service"[All Fields]) OR "school health service"[All Fields]) OR ("school health services"[MeSH Terms] OR ("school"[All Fields] AND "health"[All Fields] AND "services"[All Fields]) OR "school health services"[All Fields] OR ("service"[All Fields] AND "school"[All Fields] AND "health"[All Fields]) OR "service school health"[All Fields]) OR ("school health services"[MeSH Terms] OR ("school"[All Fields] AND "health"[All Fields] AND "services"[All Fields]) OR "school health services"[All Fields] OR ("school"[All Fields] AND "based"[All Fields] AND "health"[All Fields] AND "services"[All Fields]) OR "school based health services"[All Fields]) OR (("health services"[MeSH Terms] OR ("health"[All Fields] AND "services"[All Fields]) OR "health services"[All Fields] OR ("health"[All Fields] AND "service"[All Fields]) OR "health service"[All Fields]) AND "School-Based"[All Fields]) OR (("health services"[MeSH Terms] OR ("health"[All Fields] AND "services"[All Fields]) OR "health services"[All Fields]) AND "School-Based"[All Fields]) OR ("school health services"[MeSH Terms] OR ("school"[All Fields] AND "health"[All Fields] AND "services"[All Fields]) OR "school health services"[All Fields] OR ("school"[All Fields] AND "based"[All Fields] AND "health"[All Fields] AND "service"[All Fields]) OR "school based health service"[All Fields]) OR ("school health services"[MeSH Terms] OR ("school"[All Fields] AND "health"[All Fields] AND "services"[All Fields]) OR "school health services"[All Fields] OR ("school"[All Fields] AND "based"[All Fields] AND "health"[All Fields] AND "services"[All Fields]) OR "school based health services"[All Fields]) OR (("service"[All Fields] OR "service s"[All Fields] OR "serviced"[All Fields] OR "services"[All Fields] OR "services s"[All Fields] OR "servicing"[All Fields]) AND "School-Based"[All Fields] AND ("health"[MeSH Terms] OR "health"[All Fields] OR "health s"[All Fields] OR "healthful"[All Fields] OR "healthfulness"[All Fields] OR "healths"[All Fields])) OR (("service"[All Fields] OR "service s"[All Fields] OR "serviced"[All Fields] OR "services"[All Fields] OR "services s"[All Fields] OR "servicing"[All Fields]) AND "School-Based"[All Fields] AND ("health"[MeSH Terms] OR "health"[All Fields] OR "health s"[All Fields] OR "healthful"[All Fields] OR "healthfulness"[All Fields] OR "healths"[All Fields])) OR ("school health services"[MeSH Terms] OR ("school"[All Fields] AND "health"[All Fields] AND "services"[All Fields]) OR "school health services"[All Fields] OR ("health"[All Fields] AND "services"[All Fields] AND "school"[All Fields]) OR "health services school"[All Fields]) OR ("school health services"[MeSH Terms] OR ("school"[All Fields] AND "health"[All Fields] AND "services"[All Fields]) OR "school health services"[All Fields] OR ("services"[All Fields] AND "school"[All Fields] AND "health"[All Fields]) OR "services school health"[All Fields]) OR ("school health services"[MeSH Terms] OR ("school"[All Fields] AND "health"[All Fields] AND "services"[All Fields]) OR "school health services"[All Fields] OR ("school"[All Fields] AND "based"[All Fields] AND "services"[All Fields]) OR "school based services"[All Fields]) OR ("school health services"[MeSH Terms] OR ("school"[All Fields] AND "health"[All Fields] AND "services"[All Fields]) OR "school health services"[All Fields] OR ("school"[All Fields] AND "based"[All Fields] AND "service"[All Fields]) OR "school based service"[All Fields]) OR ("school health services"[MeSH Terms] OR ("school"[All Fields] AND "health"[All Fields] AND "services"[All Fields]) OR "school health services"[All Fields] OR ("school"[All Fields] AND "based"[All Fields] AND "services"[All Fields]) OR "school based services"[All Fields]) OR (("service"[All Fields] OR "service s"[All Fields] OR "serviced"[All Fields] OR "services"[All Fields] OR "services s"[All Fields] OR "servicing"[All Fields]) AND "School-Based"[All Fields]) OR (("service"[All Fields] OR "service s"[All Fields] OR "serviced"[All Fields] OR "services"[All Fields] OR "services s"[All Fields] OR "servicing"[All Fields]) AND "School-Based"[All Fields]) OR ("school health services"[MeSH Terms] OR ("school"[All Fields] AND "health"[All Fields] AND "services"[All Fields]) OR "school health services"[All Fields] OR ("school"[All Fields] AND "health"[All Fields] AND "promotion"[All Fields]) OR "school health promotion"[All Fields]) OR ("school health services"[MeSH Terms] OR ("school"[All Fields] AND "health"[All Fields] AND "services"[All Fields]) OR "school health services"[All Fields] OR ("health"[All Fields] AND "promotion"[All Fields] AND "school"[All Fields]) OR "health promotion school"[All Fields]) OR ("school health services"[MeSH Terms] OR ("school"[All Fields] AND "health"[All Fields] AND "services"[All Fields]) OR "school health services"[All Fields] OR ("health"[All Fields] AND "promotions"[All Fields] AND "school"[All Fields])) OR ("school health services"[MeSH Terms] OR ("school"[All Fields] AND "health"[All Fields] AND "services"[All Fields]) OR "school health services"[All Fields] OR ("promotion"[All Fields] AND "school"[All Fields] AND "health"[All Fields]) OR "promotion school health"[All Fields]) OR ("school health services"[MeSH Terms] OR ("school"[All Fields] AND "health"[All Fields] AND "services"[All Fields]) OR "school health services"[All Fields] OR ("promotions"[All Fields] AND "school"[All Fields] AND "health"[All Fields])) OR ("school health services"[MeSH Terms] OR ("school"[All Fields] AND "health"[All Fields] AND "services"[All Fields]) OR "school health services"[All Fields] OR ("school"[All Fields] AND "health"[All Fields] AND "promotions"[All Fields]))) AND ("sedentary behavior"[MeSH Terms] OR ("sedentary"[All Fields] AND "behavior"[All Fields]) OR "sedentary behavior"[All Fields] OR ("sedentary behavior"[MeSH Terms] OR ("sedentary"[All Fields] AND "behavior"[All Fields]) OR "sedentary behavior"[All Fields] OR ("behavior"[All Fields] AND "sedentary"[All Fields]) OR "behavior sedentary"[All Fields]) OR ("sedentary behavior"[MeSH Terms] OR ("sedentary"[All Fields] AND "behavior"[All Fields]) OR "sedentary behavior"[All Fields] OR ("sedentary"[All Fields] AND "behaviors"[All Fields]) OR "sedentary behaviors"[All Fields]) OR ("sedentary behavior"[MeSH Terms] OR ("sedentary"[All Fields] AND "behavior"[All Fields]) OR "sedentary behavior"[All Fields] OR ("sedentary"[All Fields] AND "lifestyle"[All Fields]) OR "sedentary lifestyle"[All Fields]) OR ("sedentary behavior"[MeSH Terms] OR ("sedentary"[All Fields] AND "behavior"[All Fields]) OR "sedentary behavior"[All Fields] OR ("lifestyle"[All Fields] AND "sedentary"[All Fields]) OR "lifestyle sedentary"[All Fields]) OR ("sedentary behavior"[MeSH Terms] OR ("sedentary"[All Fields] AND "behavior"[All Fields]) OR "sedentary behavior"[All Fields] OR ("physical"[All Fields] AND "inactivity"[All Fields]) OR "physical inactivity"[All Fields]) OR ("sedentary behavior"[MeSH Terms] OR ("sedentary"[All Fields] AND "behavior"[All Fields]) OR "sedentary behavior"[All Fields] OR ("inactivity"[All Fields] AND "physical"[All Fields]) OR "inactivity physical"[All Fields]) OR ("sedentary behavior"[MeSH Terms] OR ("sedentary"[All Fields] AND "behavior"[All Fields]) OR "sedentary behavior"[All Fields] OR ("lack"[All Fields] AND "physical"[All Fields] AND "activity"[All Fields]) OR "lack of physical activity"[All Fields]) OR ("sedentary behavior"[MeSH Terms] OR ("sedentary"[All Fields] AND "behavior"[All Fields]) OR "sedentary behavior"[All Fields] OR ("sedentary"[All Fields] AND "time"[All Fields]) OR "sedentary time"[All Fields]) OR ("sedentary behavior"[MeSH Terms] OR ("sedentary"[All Fields] AND "behavior"[All Fields]) OR "sedentary behavior"[All Fields] OR ("sedentary"[All Fields] AND "times"[All Fields]) OR "sedentary times"[All Fields]) OR ("sedentary behavior"[MeSH Terms] OR ("sedentary"[All Fields] AND "behavior"[All Fields]) OR "sedentary behavior"[All Fields] OR ("time"[All Fields] AND "sedentary"[All Fields]) OR "time sedentary"[All Fields])) AND ("exercise"[MeSH Terms] OR "exercise"[All Fields] OR ("activity"[All Fields] AND "physical"[All Fields]) OR "activity physical"[All Fields] OR ("exercise"[MeSH Terms] OR "exercise"[All Fields] OR "exercises"[All Fields] OR "exercise therapy"[MeSH Terms] OR ("exercise"[All Fields] AND "therapy"[All Fields]) OR "exercise therapy"[All Fields] OR "exercising"[All Fields] OR "exercise s"[All Fields] OR "exercised"[All Fields] OR "exerciser"[All Fields] OR "exercisers"[All Fields]) OR ("exercise"[MeSH Terms] OR "exercise"[All Fields] OR ("exercise"[All Fields] AND "physical"[All Fields]) OR "exercise physical"[All Fields]) OR ("exercise"[MeSH Terms] OR "exercise"[All Fields] OR ("exercises"[All Fields] AND "physical"[All Fields]) OR "exercises physical"[All Fields]) OR ("exercise"[MeSH Terms] OR "exercise"[All Fields] OR ("physical"[All Fields] AND "exercise"[All Fields]) OR "physical exercise"[All Fields]) OR ("exercise"[MeSH Terms] OR "exercise"[All Fields] OR ("physical"[All Fields] AND "exercises"[All Fields]) OR "physical exercises"[All Fields]) OR ("exercise"[MeSH Terms] OR "exercise"[All Fields] OR ("physical"[All Fields] AND "activity"[All Fields]) OR "physical activity"[All Fields]) OR ("exercise"[MeSH Terms] OR "exercise"[All Fields] OR ("activities"[All Fields] AND "physical"[All Fields]) OR "activities physical"[All Fields]) OR ("exercise"[MeSH Terms] OR "exercise"[All Fields] OR ("activity"[All Fields] AND "physical"[All Fields]) OR "activity physical"[All Fields]) OR ("exercise"[MeSH Terms] OR "exercise"[All Fields] OR ("physical"[All Fields] AND "activities"[All Fields]) OR "physical activities"[All Fields]) OR ("exercise"[MeSH Terms] OR "exercise"[All Fields] OR ("exercise"[All Fields] AND "aerobic"[All Fields]) OR "exercise aerobic"[All Fields]) OR ("exercise"[MeSH Terms] OR "exercise"[All Fields] OR ("aerobic"[All Fields] AND "exercise"[All Fields]) OR "aerobic exercise"[All Fields]) OR ("exercise"[MeSH Terms] OR "exercise"[All Fields] OR ("aerobic"[All Fields] AND "exercises"[All Fields]) OR "aerobic exercises"[All Fields]) OR ("exercise"[MeSH Terms] OR "exercise"[All Fields] OR ("exercises"[All Fields] AND "aerobic"[All Fields]) OR "exercises aerobic"[All Fields]) OR ("exercise"[MeSH Terms] OR "exercise"[All Fields] OR ("exercise"[All Fields] AND "isometric"[All Fields]) OR "exercise isometric"[All Fields]) OR ("exercise"[MeSH Terms] OR "exercise"[All Fields] OR ("exercises"[All Fields] AND "isometric"[All Fields]) OR "exercises isometric"[All Fields]) OR ("exercise"[MeSH Terms] OR "exercise"[All Fields] OR ("isometric"[All Fields] AND "exercises"[All Fields]) OR "isometric exercises"[All Fields]) OR ("exercise"[MeSH Terms] OR "exercise"[All Fields] OR ("isometric"[All Fields] AND "exercise"[All Fields]) OR "isometric exercise"[All Fields]) OR ("exercise"[MeSH Terms] OR "exercise"[All Fields] OR ("acute"[All Fields] AND "exercise"[All Fields]) OR "acute exercise"[All Fields]) OR ("exercise"[MeSH Terms] OR "exercise"[All Fields] OR ("acute"[All Fields] AND "exercises"[All Fields]) OR "acute exercises"[All Fields]) OR ("exercise"[MeSH Terms] OR "exercise"[All Fields] OR ("exercise"[All Fields] AND "acute"[All Fields]) OR "exercise acute"[All Fields]) OR ("exercise"[MeSH Terms] OR "exercise"[All Fields] OR ("exercises"[All Fields] AND "acute"[All Fields]) OR "exercises acute"[All Fields]) OR ("exercise"[MeSH Terms] OR "exercise"[All Fields] OR ("exercise"[All Fields] AND "training"[All Fields]) OR "exercise training"[All Fields]) OR ("exercise"[MeSH Terms] OR "exercise"[All Fields] OR ("exercise"[All Fields] AND "trainings"[All Fields]) OR "exercise trainings"[All Fields]) OR ("exercise"[MeSH Terms] OR "exercise"[All Fields] OR ("training"[All Fields] AND "exercise"[All Fields]) OR "training exercise"[All Fields]) OR ("exercise"[MeSH Terms] OR "exercise"[All Fields] OR ("trainings"[All Fields] AND "exercise"[All Fields]))) AND ("child"[MeSH Terms] OR "child"[All Fields] OR "children"[All Fields] OR "child s"[All Fields] OR "children s"[All Fields] OR "childrens"[All Fields] OR "childs"[All Fields] OR ("child"[MeSH Terms] OR "child"[All Fields] OR "children"[All Fields] OR "child s"[All Fields] OR "children s"[All Fields] OR "childrens"[All Fields] OR "childs"[All Fields]) OR ("adolescences"[All Fields] OR "adolescency"[All Fields] OR "adolescent"[MeSH Terms] OR "adolescent"[All Fields] OR "adolescence"[All Fields] OR "adolescents"[All Fields] OR "adolescent s"[All Fields]) OR ("adolescences"[All Fields] OR "adolescency"[All Fields] OR "adolescent"[MeSH Terms] OR "adolescent"[All Fields] OR "adolescence"[All Fields] OR "adolescents"[All Fields] OR "adolescent s"[All Fields]) OR ("adolescences"[All Fields] OR "adolescency"[All Fields] OR "adolescent"[MeSH Terms] OR "adolescent"[All Fields] OR "adolescence"[All Fields] OR "adolescents"[All Fields] OR "adolescent s"[All Fields]) OR ("adolescent"[MeSH Terms] OR "adolescent"[All Fields] OR ("adolescents"[All Fields] AND "female"[All Fields]) OR "adolescents female"[All Fields]) OR ("adolescent"[MeSH Terms] OR "adolescent"[All Fields] OR ("adolescent"[All Fields] AND "female"[All Fields]) OR "adolescent female"[All Fields]) OR ("adolescent"[MeSH Terms] OR "adolescent"[All Fields] OR ("female"[All Fields] AND "adolescent"[All Fields]) OR "female adolescent"[All Fields]) OR ("adolescent"[MeSH Terms] OR "adolescent"[All Fields] OR ("female"[All Fields] AND "adolescents"[All Fields]) OR "female adolescents"[All Fields]) OR ("adolescent"[MeSH Terms] OR "adolescent"[All Fields] OR ("adolescents"[All Fields] AND "male"[All Fields]) OR "adolescents male"[All Fields]) OR ("adolescent"[MeSH Terms] OR "adolescent"[All Fields] OR ("adolescent"[All Fields] AND "male"[All Fields]) OR "adolescent male"[All Fields]) OR ("adolescent"[MeSH Terms] OR "adolescent"[All Fields] OR ("male"[All Fields] AND "adolescent"[All Fields]) OR "male adolescent"[All Fields]) OR ("adolescent"[MeSH Terms] OR "adolescent"[All Fields] OR ("male"[All Fields] AND "adolescents"[All Fields]) OR "male adolescents"[All Fields]) OR ("adolescent"[MeSH Terms] OR "adolescent"[All Fields] OR "youth"[All Fields] OR "youths"[All Fields] OR "youth s"[All Fields]) OR ("adolescent"[MeSH Terms] OR "adolescent"[All Fields] OR "youth"[All Fields] OR "youths"[All Fields] OR "youth s"[All Fields]) OR ("adolescent"[MeSH Terms] OR "adolescent"[All Fields] OR "teens"[All Fields] OR "teen s"[All Fields]) OR ("adolescent"[MeSH Terms] OR "adolescent"[All Fields] OR "teen"[All Fields]) OR ("adolescent"[MeSH Terms] OR "adolescent"[All Fields] OR "teenage"[All Fields] OR "teenager"[All Fields] OR "teenagers"[All Fields] OR "teenaged"[All Fields] OR "teenager s"[All Fields] OR "teenages"[All Fields]) OR ("adolescent"[MeSH Terms] OR "adolescent"[All Fields] OR "teenage"[All Fields] OR "teenager"[All Fields] OR "teenagers"[All Fields] OR "teenaged"[All Fields] OR "teenager s"[All Fields] OR "teenages"[All Fields]))

## **Search string** for Web of Science

(TS=(Health Service, School) OR TS=(School Health Service) OR TS=(Service, School Health) OR TS=(School-Based Health Services) OR TS=(Health Service, School-Based) OR TS=(Health Services, School-Based) OR TS=(School-Based Health Service) OR TS=(School Based Health Services) OR TS=(Service, School-Based Health) OR TS=(Services, School-Based Health) OR TS=(Health Services, School) OR TS=(Services, School Health) OR TS=(School-Based Services) OR TS=(School-Based Service) OR TS=(School Based Services) OR TS=(Service, School-Based) OR TS=(Services, School-Based) OR TS=(School Health Promotion) OR TS=(Health Promotion, School) OR TS=(Health Promotions, School) OR TS=(Promotion, School Health) OR TS=(Promotions, School Health) OR TS=(School Health Promotions)) NOT (SILOID==("PPRN"))

AND

(TS=(activity, physical ) OR TS=(Exercises) OR TS=(Exercise, Physical) OR TS=(Exercises, Physical) OR TS=(Physical Exercise) OR TS=(Physical Exercises) OR TS=(Physical Activity) OR TS=(Activities, Physical) OR TS=(Activity, Physical) OR TS=(Physical Activities) OR TS=(Exercise, Aerobic) OR TS=(Aerobic Exercise) OR TS=(Aerobic Exercises) OR TS=(Exercises, Aerobic) OR TS=(Exercise, Isometric) OR TS=(Exercises, Isometric) OR TS=(Isometric Exercises) OR TS=(Isometric Exercise) OR TS=(Acute Exercise) OR TS=(Acute Exercises) OR TS=(Exercise, Acute) OR TS=(Exercises, Acute) OR TS=(Exercise Training) OR TS=(Exercise Trainings) OR TS=(Training, Exercise) OR TS=(Trainings, Exercise)) NOT (SILOID==("PPRN"))

AND

(TS=(Sedentary Behavior) OR TS=(Behavior, Sedentary) OR TS=(Sedentary Behaviors) OR TS=(Sedentary Lifestyle) OR TS=(Lifestyle, Sedentary) OR TS=(Physical Inactivity) OR TS=(Inactivity, Physical) OR TS=(Lack of Physical Activity) OR TS=(Sedentary Time) OR TS=(Sedentary Times) OR TS=(Time, Sedentary)) NOT (SILOID==("PPRN"))

AND

(TS=(Child) OR TS=(Children) OR TS=(Adolescent) OR TS=(Adolescents) OR TS=(Adolescence) OR TS=(Adolescents, Female) OR TS=(Adolescent, Female) OR TS=(Female Adolescent) OR TS=(Female Adolescents) OR TS=(Adolescents, Male) OR TS=(Adolescent, Male) OR TS=(Male Adolescent) OR TS=(Male Adolescents) OR TS=(Youth) OR TS=(Youths) OR TS=(Teens) OR TS=(Teen) OR TS=(Teenagers) OR TS=(Teenager)) NOT (SILOID==("PPRN"))

## **Search** string for SCOPUS

( ( TITLE-ABS-KEY ( health AND service, AND school ) OR TITLE-ABS-KEY ( school AND health AND service ) OR TITLE-ABS-KEY ( service, AND school AND health ) OR TITLE-ABS-KEY ( school-based health AND services ) OR TITLE-ABS-KEY ( health AND service, school-based ) OR TITLE-ABS-KEY ( health AND services, school-based ) OR TITLE-ABS-KEY ( school-based health AND service ) OR TITLE-ABS-KEY ( service, school-based health ) OR TITLE-ABS-KEY ( services, school-based health ) OR TITLE-ABS-KEY ( health AND services, AND school ) OR TITLE-ABS-KEY ( services, AND school AND health ) OR TITLE-ABS-KEY ( school-based services ) OR TITLE-ABS-KEY ( school-based service ) OR TITLE-ABS-KEY ( school AND based services ) OR TITLE-ABS-KEY ( service, school-based ) OR TITLE-ABS-KEY ( services, school-based ) OR TITLE-ABS-KEY ( school AND health AND promotion ) OR TITLE-ABS-KEY ( health AND promotion, AND school ) OR TITLE-ABS-KEY ( health AND promotions, AND school ) OR TITLE-ABS-KEY ( promotion, AND school AND health ) OR TITLE-ABS-KEY ( promotions, AND school AND health ) OR TITLE-ABS-KEY ( school AND health AND promotions ) ) ) AND ( ( TITLE-ABS-KEY ( activity, AND physical ) OR TITLE-ABS-KEY ( exercises ) OR TITLE-ABS-KEY ( exercise, AND physical ) OR TITLE-ABS-KEY ( exercises, AND physical ) OR TITLE-ABS-KEY ( physical exercise ) OR TITLE-ABS-KEY ( physical AND exercises ) OR TITLE-ABS-KEY ( physical AND activity ) OR TITLE-ABS-KEY ( activities, AND physical ) OR TITLE-ABS-KEY ( activity, AND physical ) OR TITLE-ABS-KEY ( physical AND activities ) OR TITLE-ABS-KEY ( exercise, AND aerobic ) OR TITLE-ABS-KEY ( aerobic exercise ) OR TITLE-ABS-KEY ( aerobic AND exercises ) OR TITLE-ABS-KEY ( exercises, AND aerobic ) OR TITLE-ABS-KEY ( exercise, AND isometric ) OR TITLE-ABS-KEY ( exercises, AND isometric ) OR TITLE-ABS-KEY ( isometric AND exercises ) OR TITLE-ABS-KEY ( isometric exercise ) OR TITLE-ABS-KEY ( acute exercise ) OR TITLE-ABS-KEY ( acute AND exercises ) OR TITLE-ABS-KEY ( exercise, AND acute ) OR TITLE-ABS-KEY ( exercises, AND acute ) OR TITLE-ABS-KEY ( exercise training ) OR TITLE-ABS-KEY ( exercise trainings ) OR TITLE-ABS-KEY ( training, exercise ) OR TITLE-ABS-KEY ( trainings, exercise ) ) ) AND ( ( TITLE-ABS-KEY ( sedentary AND behavior ) OR TITLE-ABS-KEY ( behavior, AND sedentary ) OR TITLE-ABS-KEY ( sedentary AND behaviors ) OR TITLE-ABS-KEY ( sedentary AND lifestyle ) OR TITLE-ABS-KEY ( lifestyle, AND sedentary ) OR TITLE-ABS-KEY ( physical AND inactivity ) OR TITLE-ABS-KEY ( inactivity, AND physical ) OR TITLE-ABS-KEY ( lack AND of physical AND activity ) OR TITLE-ABS-KEY ( sedentary AND time ) OR TITLE-ABS-KEY ( sedentary AND times ) OR TITLE-ABS-KEY ( time, AND sedentary ) ) ) AND ( ( TITLE-ABS-KEY ( child ) OR TITLE-ABS-KEY ( children ) OR TITLE-ABS-KEY ( adolescent ) OR TITLE-ABS-KEY ( adolescents ) OR TITLE-ABS-KEY ( adolescence ) OR TITLE-ABS-KEY ( adolescents, AND female ) OR TITLE-ABS-KEY ( adolescent, AND female ) OR TITLE-ABS-KEY ( female adolescent ) OR TITLE-ABS-KEY ( female AND adolescents ) OR TITLE-ABS-KEY ( adolescents, AND male ) OR TITLE-ABS-KEY ( adolescent, AND male ) OR TITLE-ABS-KEY ( male adolescent ) OR TITLE-ABS-KEY ( male AND adolescents ) OR TITLE-ABS-KEY ( youth ) OR TITLE-ABS-KEY ( youths ) OR TITLE-ABS-KEY ( teens ) OR TITLE-ABS-KEY ( teen ) OR TITLE-ABS-KEY ( teenagers ) OR TITLE-ABS-KEY ( teenager ) ) )

# Subgroup analysis of MVPA

## Age subgroup


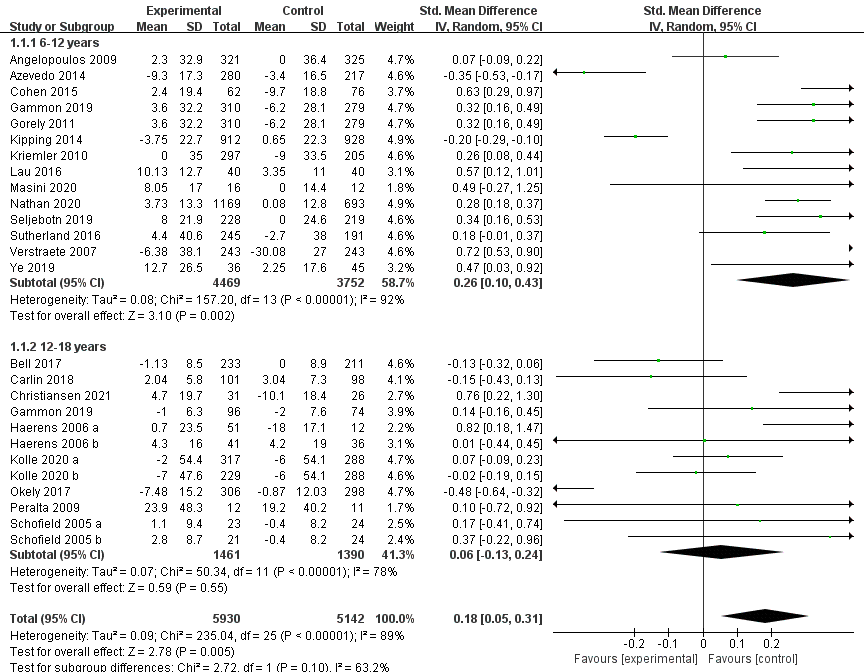


Figure. 1 Subgroup analysis of Age

## Regional subgroup


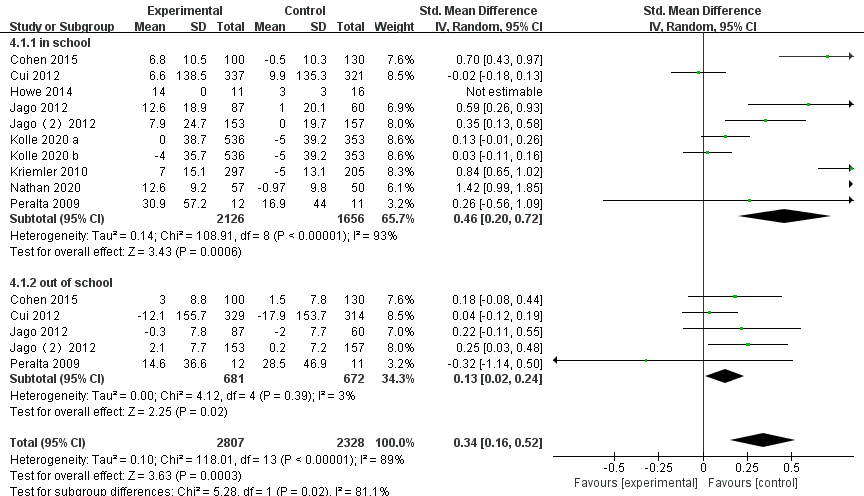


Figure. 2 Subgroup analysis of Region

## Whether or not they are in school subgroups


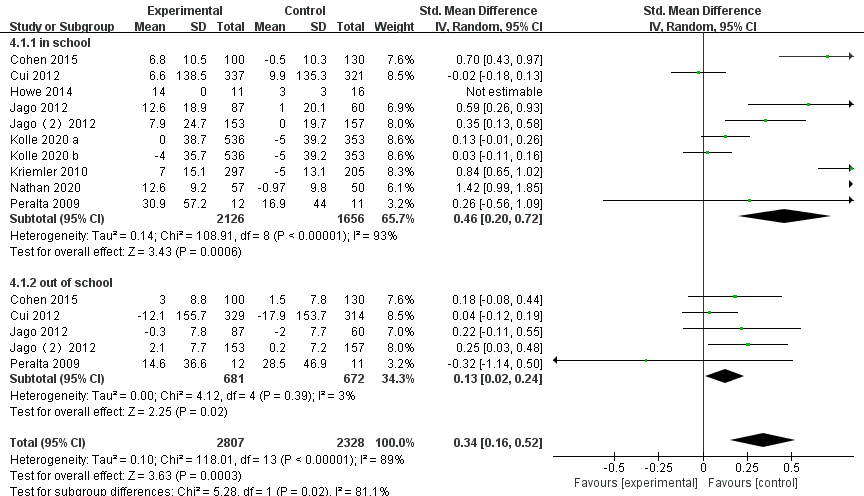


Figure. 3 Whether or not they are in school subgroups

# Detection of Publication Risk of Bias

## Egger’s test and Begg’s test for MVPA

Figure. 4 Egger’s test for MVPA

Figure. 5 Begg’s test for MVPA

## Egger’s test and Begg’s test for ST

Figure. 6 Egger’s test for ST

Figure. 7 Egger’s test for ST

## Egger’s test and Begg’s test for LPA

Figure. 8 Egger’s test for LPA

Figure. 9 Egger’s test for LPA
